# Complete human serum maintains viability and chondrogenic potential of human synovial stem cells: suitable conditions for transplantation

**DOI:** 10.1186/s13287-017-0596-0

**Published:** 2017-06-13

**Authors:** Mitsuru Mizuno, Hisako Katano, Koji Otabe, Keiichiro Komori, Yuji Kohno, Shizuka Fujii, Nobutake Ozeki, Masafumi Horie, Kunikazu Tsuji, Hideyuki Koga, Takeshi Muneta, Ichiro Sekiya

**Affiliations:** 1 Center for Stem Cell and Regenerative Medicine, Tokyo Medical and Dental University, 1-5-45 Yushima, Bunkyo-ku, Tokyo, 113-8510 Japan; 2 Department of Joint Surgery and Sports Medicine, Graduate School, Tokyo Medical and Dental University, Tokyo, Japan; 3 Department of Cartilage Regeneration, Graduate School, Tokyo Medical and Dental University, Tokyo, Japan; 4 National Hospital Organization Disaster Medical Center, Tokyo, Japan

**Keywords:** Human serum, Mesenchymal stem cells, Synovium, Regenerative medicine, Cell preservation

## Abstract

**Background:**

In our clinical practice, we perform transplantations of autologous synovial mesenchymal stem cells (MSCs) for cartilage and meniscus regenerative medicine. One of the most important issues to ensuring clinical efficacy involves the transport of synovial MSCs from the processing facility to the clinic. Complete human serum (100% human serum) is an attractive candidate material in which to suspend synovial MSCs for their preservation during transport. The purpose of this study was to investigate whether complete human serum maintained MSC viability and chondrogenic potential and to examine the optimal temperature conditions for the preservation of human synovial MSCs.

**Methods:**

Human synovium was harvested from the knees of 14 donors with osteoarthritis during total knee arthroplasty. Passage 2 synovial MSCs were suspended at 2 million cells/100 μL in Ringer’s solution or complete human serum at 4, 13, and 37 °C for 48 h. These cells were analyzed for live cell rates, cell surface marker expression, metabolic activity, proliferation, and adipogenic, calcification, and chondrogenic differentiation potentials before and after preservation.

**Results:**

After preservation, synovial MSCs maintained higher live cell rates in human serum than in Ringer’s solution at 4 and 13 °C. Synovial MSCs preserved in human serum at 4 and 13 °C also maintained high ratios of propidium iodide^–^ and annexin V^–^ cells. MSC surface marker expression was not altered in cells preserved at 4 and 13 °C. The metabolic activities of cells preserved in human serum at 4 and 13 °C was maintained, while significantly reduced in other conditions. Replated MSCs retained their proliferation ability when preserved in human serum at 4 and 13 °C. Adipogenesis and calcification potential could be observed in cells preserved in each condition, whereas chondrogenic potential was retained only in cells preserved in human serum at 4 and 13 °C.

**Conclusion:**

The viability and chondrogenic potential of synovial MSCs were maintained when the cells were suspended in human serum at 4 and 13 °C.

**Electronic supplementary material:**

The online version of this article (doi:10.1186/s13287-017-0596-0) contains supplementary material, which is available to authorized users.

## Background

**Fig. 1:**
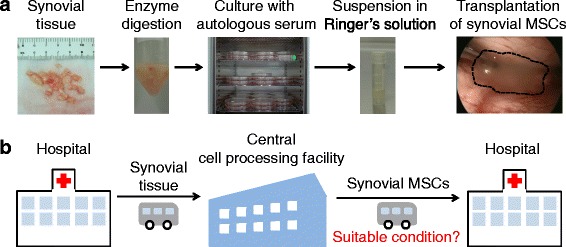
Scheme for regenerative medicine using synovial mesenchymal stem cells (*MSCs*). **a** Procedure for cartilage and meniscus regenerative medicine with autologous synovial MSCs. **b** Outline of transportation of synovial tissue and synovial MSCs between a hospital and a central cell-processing facility. Suitable conditions for transporting synovial MSCs from the central cell-processing facility to a hospital are essential

It has been shown that mesenchymal stem cells (MSCs) derived from synovium readily expand in vitro in human serum and differentiate into cartilage and meniscus tissue when transplanted into these defects in vivo [1, 2]. We have clinically transplanted autologous synovial MSCs for the therapeutic regeneration of articular cartilage and the meniscus. For the procedure, synovial tissue was harvested arthroscopically and enzymatically digested, synovial cells were cultured with 10% autologous human serum for 14 days, and then synovial MSCs were suspended in 0.5 mL glucose acetate Ringer’s solution and transplanted arthroscopically onto the cartilage and/or meniscus defects (Fig. 1a). Cell culture was performed in the cell processing facility at our hospital.

Synovial MSCs must be prepared at a central cell-processing facility to produce enough autologous synovial MSCs for regenerative medicine applications. Synovial tissue was transferred from a hospital to a central cell-processing facility, the synovial MSCs were prepared there, and then synovial MSCs were transferred back to the hospital (Fig. 1b). The handling of the cells during transport is of particular importance, as this is the last major step before implantation to patients. Here, we wanted to determine the best suspension conditions, temperature, and medium for the transport of synovial MSCs to guarantee patient safety.

Human serum, the plasma component of blood that lacks coagulation factors, is similar to interstitial fluid and can provide all of the basic components necessary for normal cell functions [3]. The most abundant protein in human serum is albumin which constitutes approximately one-half of the blood serum protein, buffers pH, and maintains osmotic pressures [4]. Therefore, human serum is a candidate material to suspend synovial MSCs for their preservation during transport. The purpose of this study was to investigate whether complete human serum (100% human serum) maintained viability and chondrogenic potential and to examine the optimal temperature for the 48-h preservation of human synovial MSCs.

## Methods

### Collection of human serum

The present study was approved by the Medical Research Ethics Committee of Tokyo Medical and Dental University (approval no. 2121) and all study subjects provided informed consent. One hundred milliliters of fresh blood was collected from four healthy female volunteers (21–22 years of age) using a closed bag system (JMS Co. Ltd., Hiroshima, Japan) [5, 6]. The bag containing glass beads was shaken at room temperature for 30 min, and then the serum was separated. The serum was filtered through a 0.45-μm nylon filter (Thermo Fisher Scientific, MA, USA) and stored at –20 °C until use.

### Synovial MSCs

Human synovium was harvested from the knees of 14 donors (65–80 years of age) with osteoarthritis during total knee arthroplasty. Synovium was digested in a solution of 3 mg/mL collagenase (Sigma-Aldrich Japan, Tokyo, Japan) at 37 °C. After 3 h, the digested cells were filtered through a 70-μm cell strainer (Greiner Bio-One GmbH, Frickenhausen, Germany). The cells were cultured in α-minimum essential medium (α-MEM; Thermo Fisher Scientific) supplemented with 1% antibiotic-antimycotic (Thermo Fisher Scientific) and 10% fetal bovine serum (FBS) in a cell culture incubator (Astec Co. Ltd., Fukuoka, Japan) at 37 °C under 5% CO_2_. These cells were counted via an automated cell counter (Luna-FL, Logos Biosystems, VA, USA) with a disposable cell counting plate to determine the number of nucleated cells.

### Cell preservation

**Fig. 2:**
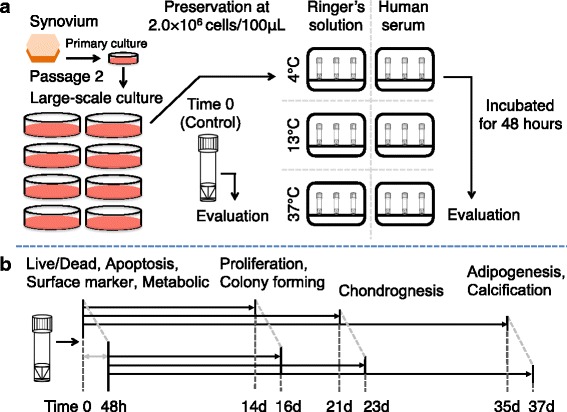
Scheme of the present study. **a** Two million passage 2 human synovial MSCs were suspended in glucose acetated Ringer’s solution or complete human serum. The cells were preserved at 4 °C, 13 °C, or 37 °C for 48 h, and then the cells were evaluated. **b** The cells were analyzed at time 0 and 48 h after preservation for live/dead, apoptosis, surface marker and metabolic assays. Then, the cells were cultured for 14 days for proliferation and colony forming abilities. The cells were also pelleted and cultured in chondrogenic medium for 21 days. Furthermore, the cells were cultured for 14 days to make cell colonies, then cultured in adipogenic medium and in calcification medium for 21 days

Synovial MSCs at passage 2 were harvested using TrypLE™ Select (Thermo Fisher Scientific). Two million synovial MSCs were suspended in 100 μL glucose acetated Ringer’s solution (KYOWA CritiCare Co. Ltd., Tokyo, Japan) as a control, or in human serum with a preservation tube (Sumitomo Bakelite Co. Ltd., Akita, Japan). The cells were incubated for 48 h at 4 °C in a refrigerator, 13 °C in a cool incubator, or 37 °C in a cell culture incubator (Fig. 2a). Then, cell evaluations were performed according to the experimental design (Fig. 2b).

### Live cell rate and cell size

Synovial MSCs at passage 2 from three donors were double-stained by acridine orange (AO) for live cells and propidium iodide (PI) for dead/apoptotic cells with a live/dead assay kit (Logos Biosystems). Live cell rate and cell size were analyzed by fluorescence imaging with Luna-FL following the manufacturer’s protocol.

### Cell apoptosis

To assess loss of membrane integrity for synovial MSCs, flow cytometry was used with an Annexin V Detection Kit 2 (Becton, Dickinson and Company, BD, NJ, USA) by a FACS Verse system (BD). Briefly, cells were suspended in 1× binding buffer at a density of 1 × 10^6^ cells/mL and stained for 15 min at room temperature with the fluorescein isothiocinate (FITC) annexin-V and PI solution in the dark. PI^–^ FITC^–^ cells were evaluated as live cells. These data were analyzed using FlowJo software (Tree Star Inc., CA, USA).

### Flow cytometric analysis of surface markers

Surface marker expression was analyzed by FACS Verse (BD) in synovial MSCs from four donors at passage 2. The cells before and 48 h after preservation were suspended in Hank’s balanced salt solution (HBSS) at a density of 5 × 10^5^ cells/mL and stained for 30 min on ice with the antibodies CD44-PE-Cy7, CD73-V450, CD90-PE, CD105-APC, CD34-PE-Cy5, CD45-APC-H7, and CD31-FITC (all from BD), and Ghost Dye Violet 510 for dead cells (Tonbo Biosciences, CA, USA). These data were also analyzed using FlowJo software.

### Metabolic assay

Synovial MSCs from four donors at passage 2 before and after preservation were analyzed for metabolic activity. For cellular dehydrogenase activity, the cells were suspended in α-MEM at a density of 1 × 10^5^ cells and were reacted with the working solution for 30 min at 37 °C and quantified by WST-8 assay (Dojindo, Kumamoto, Japan). The supernatant of cell products before and after preservation was also reacted at room temperature and quantified by lactate dehydrogenase (LDH) assay (Dojindo). Absorbance of the metabolized solution was measured by a plate reader (Infinite M200; Tecan, Männedorf, Switzerland).

### Fold increase in cell number and colony formation assay

For fold-increase assays, synovial MSCs at passage 2 from three donors were plated at 1.0 × 10^4^ cells/60 cm^2^. For cells 48 h after preservation, acridine orange-positive cells were re-counted as living cells and plated on dishes at 1.0 × 10^4^ cells/60 cm^2^. The cells were harvested with 0.25% trypsin and 1 mM ethylenediaminetetraacetic acid (EDTA; Thermo Fisher Scientific) at 37 °C for 5 min and counted with a cell counting plate.

For colony formation assays, synovial MSCs at passage 2 were also plated at 1.0 × 10^4^ cells/60 cm^2^. The dishes were stained with 1% crystal violet at 14 days. To compare the number of colonies among three dishes from three donors, one representative dish was shown, and the numbers of colonies were counted by the other three independent observers (MM, SF, and KK).

### Differentiation assays

For adipogenesis, synovial MSCs were plated at 100 cells/60-cm^2^ dish and cultured for 14 days in culture medium. The adherent cells were cultured in adipogenic induction medium supplemented with 100 nM dexamethasone, 0.5 mM isobutyl-methylxanthine (Sigma-Aldrich), and 50 mM indomethacin (Wako) for an additional 21 days. Adipocytes were stained with oil red-o staining (Muto Pure Chemicals, Tokyo, Japan).

For calcification, 100 cells were transferred to a 60-cm^2^ dish and cultured for 14 days in culture medium. The adherent cells were cultured in calcification induction medium containing 50 μg/mL ascorbic acid 2-phosphate (Wako), 10 nM dexamethasone (Wako), and 10 mM β-glycerophosphate (Sigma-Aldrich). After 21 days, calcification was assessed by alizarin red staining (Merck Millipore, Billerica, MA, USA).

For chondrogenesis, 250,000 synovial MSCs were transferred to a 15-mL tube (BD Falcon) and cultured in chondrogenic induction medium containing 10 ng/mL transforming growth factor-β3 (Miltenyi Biotec K.K., Tokyo, Japan) and 1 μg/mL bone morphogenetic protein 2 (Medtronic, TN, USA), which was changed every 3–4 days. After 21 days, cartilage pellets were weighed and evaluated histologically.

### Statistical analysis

All data were statistically evaluated by analysis of variance with GraphPad Prism 6 (GraphPad Software, CA, USA). Data are expressed as median ± interquartile range (IQR). Each statistical analysis method is described in the figure legend. Two-tailed *p* values of < .05 were considered to be significant.

## Results

### Live cell rate and size of synovial MSCs 48 h after preservation

**Fig. 3:**
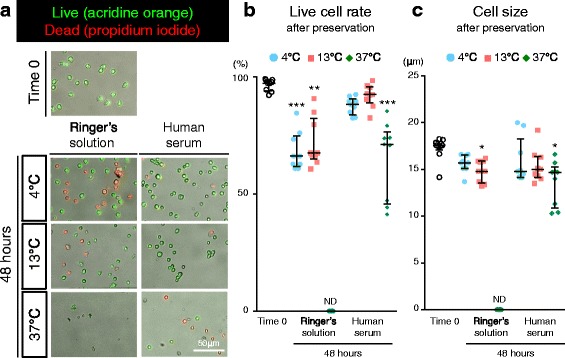
Live cell rate and size of synovial MSCs before and 48 h after preservation. **a** Representative images of live cells stained by AO (*green*) and dead cells stained by PI (*red*). **b** Live cell rate. **c** Cell size analyzed from fluorescence imaging. Median values and interquartile ranges are shown (*n* = 3). *p < .05, **p < .01, ***p < .001, compared with the value at Time 0 by Friedman test followed by Dunn’s multiple comparisons. *ND* not detected

After staining for the live cell rate, most synovial MSCs were green (viable) at time 0, while more cells were red (nonviable) 48 h after preservation except cells preserved in Ringer’s solution at 37 °C, in which normal cell morphologies were hardly detectable (Fig. 3a). The live cell rate significantly decreased in the cells preserved in Ringer’s solution at each temperature and in human serum at 37 °C, while there was no significant decrease in the live cell rate for cells preserved in human serum at 4 and 13 °C (Fig. 3b). Cell size, analyzed from the fluorescence images, was not affected after preservation in Ringer’s solution at 4 °C and human serum at 4 and 13 °C (Fig. 3c).

### Apoptosis of synovial MSCs 48 hours after preservation

**Fig. 4:**
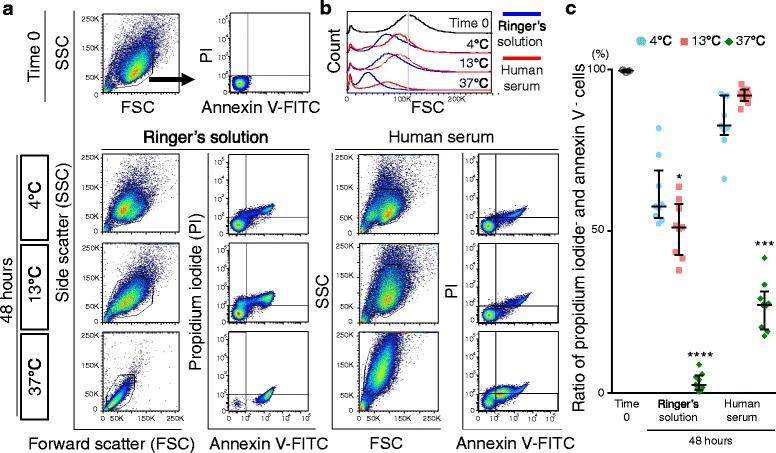
Apoptosis of synovial MSCs 48 h after preservation. **a** Representative profiles of synovial MSCs by forward scatter (*FSC*) and side scatter (*SSC*), stained with fluorescein isothiocinate (*FITC*)-annexin V and propidium iodide (*PI*). Gates were placed around major cell populations. **b** Representative forward scatter histograms. **c** Apoptotic to normal cell ratios. Cells negative for annexin V and propidium iodide were considered nonapoptotic cells. Median values and interquartile ranges are shown (*n* = 3). *p < .05, ***p < .001, ****p < .0001, compared with the value at Time 0 by Kruskal-wallis test followed by Dunn’s multiple comparisons

According to forward scatter (FSC) analyses, synovial MSCs appeared to shift to a smaller size profile 48 h after preservation, particularly cells preserved at 37 °C (Fig. 4a and b). Profiles of cells by forward scatter and side scatter (SSC) indicated that the two populations were present, especially in the cells 48 h after preservation at 4 °C; the major population of the cells preserved in Ringer’s solution was located on the left, while those in human serum were located on the right (Fig. 4a). The population on the left was strongly stained by propidium iodide, while the population on the right was not stained by propidium iodide (Additional file 1: Figure S1).

The ratio of propidium iodide^+^ and annexin V^+^ cells, classified as late apoptotic, increased in the cells 48 h after preservation in Ringer’s solution at 4 °C and 13 °C (Additional file 2: Figure S2 and Additional file 3: Table S1). The ratio of propidium iodide^+^ and annexin V^+^ cells increased after preservation in human serum at 37 °C (Additional file 2: Figure S2 and Additional file 3: Table S1). The ratio of propidium iodide^–^ and annexin V^–^ cells, classified as live cells, decreased significantly after preservation in Ringer’s solution at 13 and 37 °C and in human serum at 37 °C, while the ratio was maintained in the cells preserved in human serum at 4 and 13 °C (Fig. 4c).

### Surface markers of synovial MSCs before and 48 h after preservation

**Fig. 5:**
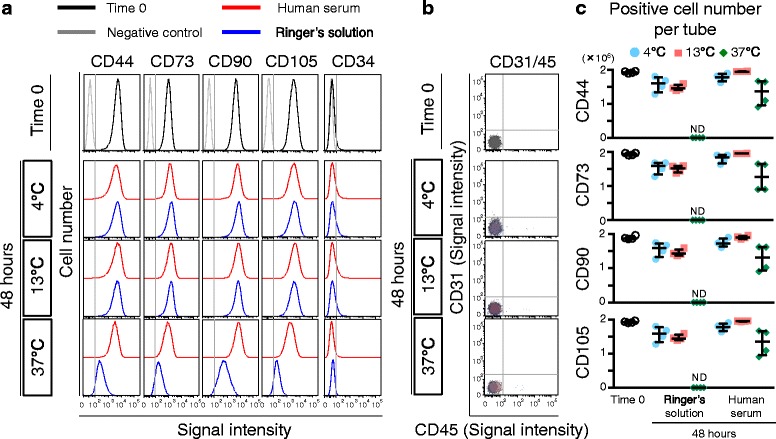
Surface markers of synovial MSCs before and 48 h after preservation. **a** Representative histogram of synovial MSCs by CD44-PE-Cy7, CD73-V450, CD90-PE, CD105-APC, and CD34-PE-Cy5. **b** Representative dot plot of synovial MSCs by CD31-FITC and CD45-APC-H7 as negative markers. **c** Calculated live cell number per tube (2 million synovial MSCs). Median values and interquartile ranges are shown (*n* = 4). *ND* not detected

Passage 2 synovial MSCs before and 48 h after preservation were stained with antibodies for MSC markers. In synovial MSCs before preservation, positive rates for CD44, CD73, CD90, and CD105 were higher than 90%, CD34 was approximately 10% (Fig. 5a), and those for CD31 and CD45 were less than 1% (Fig. 5b). In the cells 48 h after preservation, positive rates for these markers were comparable to those in synovial MSCs at time 0, except in the cells 48 h after preservation at 37 °C in Ringer’s. Positive cell numbers for CD44, 73, 90, and 105 showed no obvious differences between experimental groups, except in the cells 48 h after preservation at 37 °C in Ringer’s (Fig. 5c).

### Metabolic activity of synovial MSCs before and 48 h after preservation

**Fig. 6:**
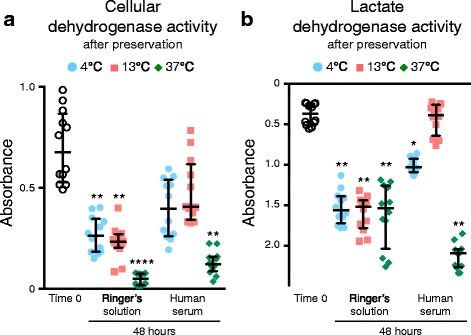
Metabolic activity of synovial MSCs before and 48 h after preservation. Passage 2 synovial MSCs before and 48 h after preservation were analyzed by colorimetric assay. **a** Cellular dehydrogenase activity as live cell metabolic activity. **b** Lactate dehydrogenase activity as dead cell metabolic activity. Median values and interquartile ranges are shown (*n* = 4). *p < .05, **p < .01, ****p < .001, compared with the value at Time 0 by Friedman test followed by Dunn’s multiple comparisons

Cellular dehydrogenase activity for live cell metabolism and lactate dehydrogenase activity indicating destruction of cell membranes was examined in passage 2 synovial MSCs before and 48 h after preservation (Fig. 6). Cellular dehydrogenase activity was maintained in cells 48 h after preservation in human serum at 4 °C and 13 °C, while it was significantly reduced under the other conditions (Fig. 6a). Lactate dehydrogenase activity was maintained in cells 48 h after preservation in human serum at 13 °C, while it was significantly decreased under the other conditions (Fig. 6b).

### Fold increase in cell number and colony formation ability of synovial MSCs 48 h after preservation

**Fig. 7:**
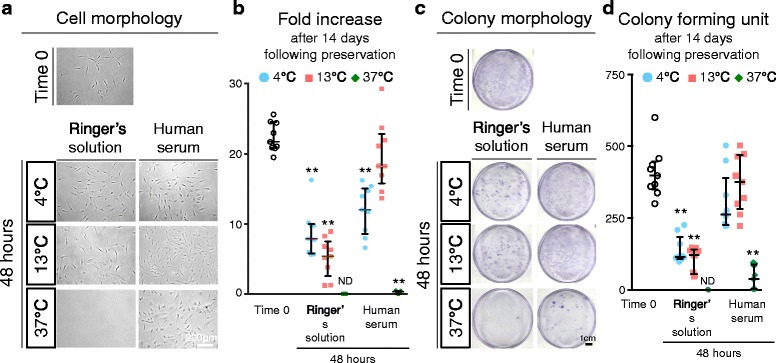
Fold increase and colony formation unit of synovial MSCs before and 48 h after preservation. Passage 2 synovial MSCs before and 48 h after preservation were replated and cultured for 14 days. **a** Representative cell morphologies of synovial MSCs. **b** Fold increases after 14 days in culture before and 48 h after preservation. Median values and interquartile ranges are shown (*n* = 3). **c** Representative dishes stained with crystal violet. **d** Colony forming units after 14 days in culture before and 48 h after preservation. Median values and interquartile ranges are shown (*n* = 3). **p < .01, compared with the value at Time 0 by Friedman test followed by Steel’s multiple comparisons. *ND* not detected

Passage 2 synovial MSCs before and 48 h after preservation were stained with a live/dead assay kit, live cells (green) were plated at 1.0 × 10^4^ cells/60 cm^2^, and cultured for 14 days. No obvious morphological differences were observed in synovial MSCs irrespective of preservation condition (Fig. 7a). Fold increase in the number of cells after 14 days in culture following the 48 h after preservation in human serum at 13 °C was comparable to the cells at time 0, while that of cells preserved under the other conditions was significantly lower than that of the cells at time 0 (Fig. 7b). When cells were plated at the same density, the number of colonies was much less in cells from 48 h after preservation at 37 °C (Fig. 7c). Colony number significantly decreased in cells 48 h after preservation in Ringer’s solution at each temperature, and in human serum at 37 °C (Fig. 7d).

### Adipogenesis and calcification of synovial MSCs before and 48 h after preservation

**Fig. 8:**
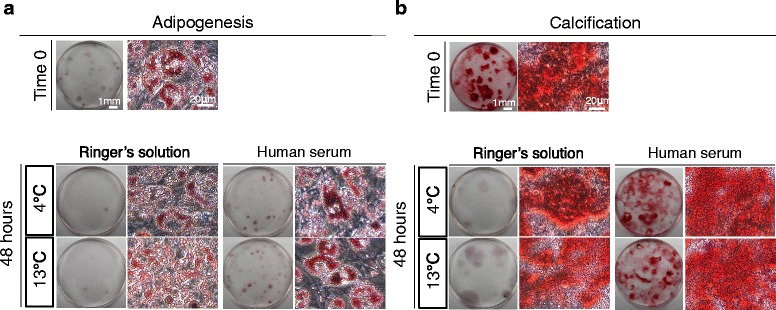
Adipogenesis and calcification of synovial MSCs before and 48 h after preservation. Passage 2 synovial MSCs before and 48 h after preservation were cultured in differentiation medium for 21 days after colony formation. **a** Representative culture dishes and cell morphology stained with oil red-o after adipogenic differentiation. **b** Representative culture dishes and cell morphology stained with alizarin red after calcification

After adipogenic induction, the synovial MSCs contained lipid, shown as red after oil red-o staining, regardless of preservation (Fig. 8a). The number of oil red-o-positive colonies appeared to decrease in cells 48 h after preservation in Ringer’s solution at 4 °C and 13 °C, while maintenance of cells 48 h after preservation in human serum was improved at each temperature.

After calcification induction, alizarin red-positive matrix was observed in cells regardless of preservation (Fig. 8b). The number of alizarin red-positive colonies appeared to decrease in cells 48 h after preservation in Ringer’s solution at 4 °C and 13 °C, while maintenance of cells 48 h after preservation in human serum was improved at each temperature.

### Chondrogenic ability of synovial MSCs 48 h after preservation

**Fig. 9:**
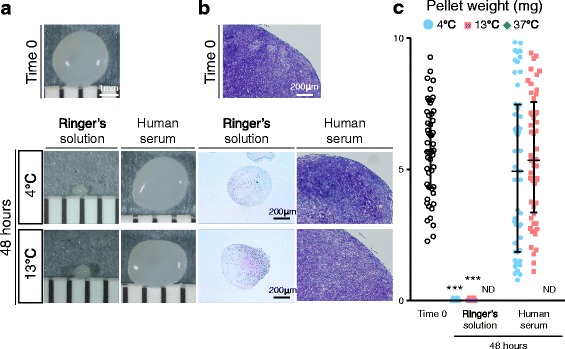
Chondrogenic ability of synovial MSCs before and 48 h after preservation. Passage 2 synovial MSCs before and 48 h after preservation were pelleted and cultured in chondrogenic medium for 21 days. **a** Representative macroscopic images of cartilage pellets. **b** Histological sections stained with toluidine blue. **c** Wet weight of cartilage pellets. Median values and interquartile ranges are shown (*n* = 10). ***p < .001, compared with the value at Time 0 by Kruskal-Wallis test followed by Dunn’s multiple comparisons. ND not detected

The size of cartilage pellets for cells at time 0 was comparable to that of cells differentiated 48 h after preservation in human serum at 4 °C and 13 °C, while the size of cartilage pellets in cells differentiated 48 h after preservation in Ringer’s solution at 4 °C and 13 °C decreased (Fig. 9a). During the in vitro chondrogenesis of cells differentiated 48 h after preservation at 37 °C, the cells did not condense (Additional file 4: Figure S3) and failed to differentiate into cartilage. Histological sections stained with toluidine blue were purple in each condition, verifying the existence of proteoglycans (Fig. 9b). In comparison with the cells at time 0, the wet weights of cartilage pellets were comparable to the cells differentiated 48 h after preservation in human serum at 4 °C and 13 °C, and were significantly decreased or absent in the cells preserved under the other conditions (Fig. 9c). Coefficient of variation for pellet weights of the cells differentiated before preservation was 31%, whereas for the cells differentiated 48 h after preservation in human serum at 13 °C it was 44%, while that at 4 °C was 61%, indicating lower pellet weight variability in the cells preserved at 13 °C than at 4 °C, though this was still higher than before preservation (Additional file 3: Table S2).

## Discussion

We investigated whether complete human serum maintained the viability and chondrogenic potential, and examined the ideal temperature for the 48-h preservation, of human synovial MSCs. When synovial MSCs were preserved in human serum at 13 °C, live cell rates, apoptosis rates, surface markers, metabolic activities, proliferation potentials, and chondrogenic potentials of the cells were maintained. When synovial MSCs were preserved in human serum at 4 °C these were also maintained, except for lactate dehydrogenase activity and cell number fold increase.

The wet weight of cartilage pellets in the cells 48 h after preservation in human serum at 4 and 13 °C was comparable to the cells at time 0. However, pellet weights in the cells preserved at 4 °C varied more than at 13 °C. Preservation at 13 °C in human serum was better with regards to maintenance of cell viabilities and chondrogenic abilities than at 4 °C or 37 °C. A temperature of 37 °C is suitable for cell culture, as it maximizes metabolic activity; however, this depletes nutrients in human serum at an early phase. Meanwhile, low temperatures reduce the metabolic activity of cells; however, excess low temperatures are harmful for maintaining the viability of the cells [7, 8]. In the case of cell shipment, it would be easier to transport cells in a certain temperature zone rather than at a specific temperature. From our results, a temperature zone between 4 and 13 °C is appropriate for transplantation of synovial MSCs.

In this study, glucose acetated Ringer’s solution was used as a control for the preservation of synovial MSCs since, in our previous clinical study, synovial MSCs were suspended in glucose acetated Ringer’s solution and this suspension was directly placed in the cartilage defect in the knee for cartilage regeneration therapy [1]. Ringer's solution is a solution of several salts dissolved in water for the purpose of creating an isotonic solution relative to bodily fluids and is nontoxic to most cells; however, it is not an ideal solution for the preservation of cells. The demonstration of advantages of human serum over Ringer’s solution was important to us.

Complete human serum maintained the viability and chondrogenic potential of human synovial MSCs. In addition to a large amount of albumin, human serum contains various cytokines, such as platelet-derived growth factors (PDGFs) which have been shown to improve cell metabolic activity in other studies [9, 10]. In clinical practice, autologous human serum is used as eye drops for ocular surface disorders [11, 12].

FBS may have the same effect as human serum for cell preservation. However, FBS has disadvantages, possibly because of pathogen transmission and immune reaction. Autologous human serum is more useful than FBS for cell preservation in the clinical situation from a safety point of view.

To preserve cells, cryopreservation is a popular method; however, it has some problems. First, cryopreservation induces formation of ice crystals, causing rupture of the cell membrane [13] which results in decreased cell viability. Second, dimethyl sulfoxide (DMSO) is widely added to the cryopreserved solution, although DMSO shows toxicity. Before transplantation of cells preserved in frozen medium containing DMSO, the DMSO must be diluted away in a cell-processing facility. For this procedure, a clean room environment is required which becomes a major obstacle to its widespread use in regenerative medicine. This also increases manufacturing costs. For the preservation of cells, some alternatives to freezing have been reported, such as green tea polyphenol, also called as epigallocatechin-3-gallate (EGCG) [14, 15]. However, this solution has not been approved for pharmaceutical use in humans.

We propose three limitations in this study. First, we used FBS instead of human serum for the isolation and proliferation of synovial MSCs because this study needed approximately forty million synovial MSCs for each donor and nearly 3000 mL serum was required for the experiments for all donors, which made it difficult to complete this study with human serum only. We previously reported that chondrogenic ability in vitro and cartilage regeneration ability in vivo were comparable in synovial MSCs cultured with FBS or human serum [16]. Though FBS may have some effect on the synovial MSCs in this study, we think our conclusion is still of value to the field of regenerative medicine for cartilage repair.

Second, we used allogeneic serum instead of autologous serum to preserve synovial MSCs. In this preclinical study, synovium was harvested from patients during knee surgery; therefore, to prepare autologous human serum was practically difficult for this study. Arpornmaeklong et al. reported that growth and osteogenic differentiation of human periodontal ligament MSCs cultured with allogenic human serum were the same as those cultured with autologous human serum [17], suggesting that allogenic human serum and autologous human serum were comparable for the proliferation and differentiation of MSCs derived from other mesenchymal tissues. In clinical practice, we will isolate and expand synovial MSCs only with autologous human serum and transplant primary synovial MSCs for cartilage and meniscus regeneration, as reported before [1], because autologous human serum is safer than allogenic human serum from the standpoint of immune reaction and disease transmission.

Third, the donors for serum were young and healthy, though the elderly are included as candidates for regenerative medicine for cartilage and meniscus therapies. There may be potential issues for the planned clinical use where autologous serum is derived from older individuals who may have health issues and whose serum may not be suitable to preserve their synovial MSCs. However, individuals in poor general condition will be excluded for this treatment. For preservation of cells, possible serum factors influenced by aging are albumin concentration, pH, and osmotic pressure. Among these, albumin concentration in serum decreases with aging; however, according to the 2013 National Health and Nutrition Survey in Japan, serum albumin concentration in persons in their 60s was not lowered by more than 10% more than in persons in their 20s, both males and females, suggesting aging may not influence serum for the preservation of cells to a great extent.

The viability and chondrogenic potential of synovial MSCs was maintained when the cells were suspended in complete human serum. Human serum is similar to interstitial fluid, which is essential for normal cell function, and contains abundant albumin, which buffers pH and maintains osmotic pressures. Synovial MSCs suspended in complete autologous human serum can be injected directly into the knee joint as it should not pose any safety concerns.

## Conclusions

Synovial MSCs suspended in complete human serum and preserved between 4 and 13 °C maintained their viability and chondrogenic potential after 48 h.

## Additional files

Additional file 1: Figure S1. Dot plot of synovial MSCs 48 h after preservation in Ringer’s solution and histograms of synovial MSCs by propidium iodide in two fractions by FSC and FFC. Two fractions were observed by FSC and FFC in cells 48 h after preservation in Ringer’s solution. The left fraction had high a PI-positive rate and the right fraction had a low PI-positive rate. (PDF 349 kb)

Additional file 2: Figure S2. Ratio of propidium iodide positive and annexin V positive synovial MSCs (late apoptotic state). Synovial MSCs before and 48 h after preservation in Ringer’s solution and human serum at each temperature were examined. *p < .05, **p < .01, compared with the value at Time 0 by Friedman test followed by Steel’s multiple comparisons. (PDF 50 kb)

Additional file 3: Table S1. Ratio of propidium iodide +/– and annexin V +/– synovial MSCs. Synovial MSCs before and 48 h after preservation in Ringer’s solution and human serum at each temperature were examined (*n* = 9). **Table S2.** Wet weight of cartilage pellets derived synovial MSCs. Synovial MSCs before and 48 h after preservation in Ringer’s solution and human serum at each temperature were examined (*n* = 60). (DOCX 21 kb)

Additional file 4: Figure S3. In vitro cartilage formation by pellet culture of synovial MSCs. Synovial MSCs before and 48 h after preservation in human serum at each temperature were pelleted after centrifugation, then cell pellets were cultured in chondrogenic medium for 21 days. Spheroids of cartilage derived from synovial MSCs were observed in cells preserved at 4 and 13 °C, while synovial MSCs were not condensed in cells preserved at 37 °C. Dot line shows cell pellet. (PDF 332 kb)

## Abbreviations

AO: Acridine orange
DMSO: Dimethyl sulfoxide
EDTA: Ethylenediaminetetraacetic acid
FACS: Fluorescence-activated cell sorter
FBS: Fetal bovine serum
FITC: Fluorescein isothiocinate
FSC: Forward scatter
LDH: Lactate dehydrogenase
MEM: Minimum essential medium
MSC: Mesenchymal stem cell
PDGF: Platelet-derived growth factor
PI: Propidium iodide
SSC: Side scatter
WST-8: 2-(2-methoxy-4-nitrophenyl)-3-(4-nitrophenyl)-5-(2,4-disulfophenyl)-2H tetrazolium, monosodium salt
