# Supplementary figures and images for: Complete human serum maintains viability and chondrogenic potential of human synovial stem cells: suitable conditions for transplantation
Source: Stem Cell Res Ther. 2017 Jun 13;8:144. doi: 10.1186/s13287-017-0596-0 (PMC5470274; doi:10.1186/s13287-017-0596-0)

Supplementary figure 1

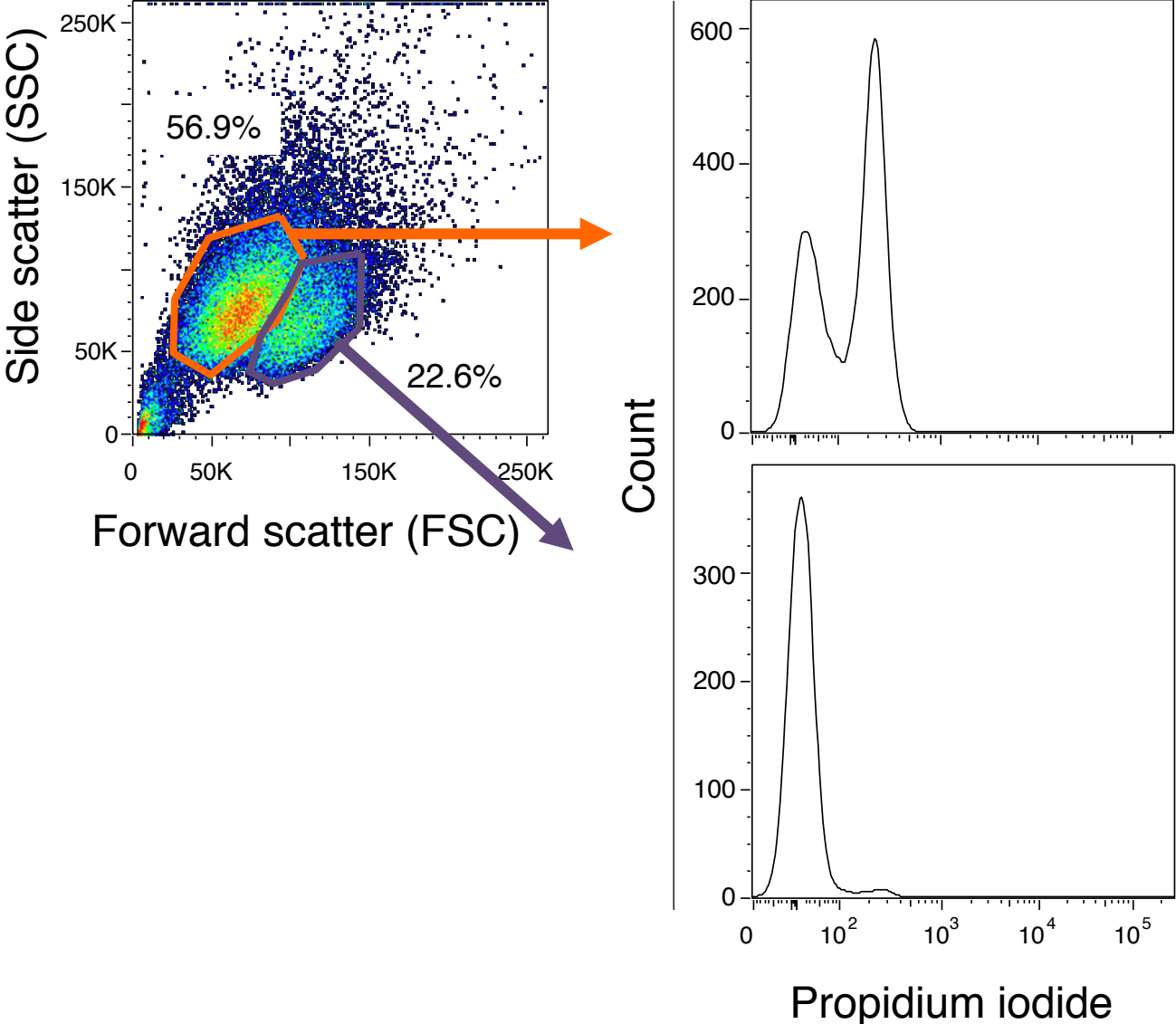

Supplement: Supplementary file 1 — Dot plot of synovial MSCs 48 h after preservation in Ringer’s solution and histograms of synovial MSCs by propidium iodide in two fractions by FSC and FFC. Two fractions were observed by FSC and FFC in cells 48 h after preservation in Ringer’s solution. The left fraction had high a PI-positive rate and the right fraction had a low PI-positive rate. (PDF 349 kb) [file 13287_2017_596_MOESM1_ESM.pdf]

Supplementary figure 2

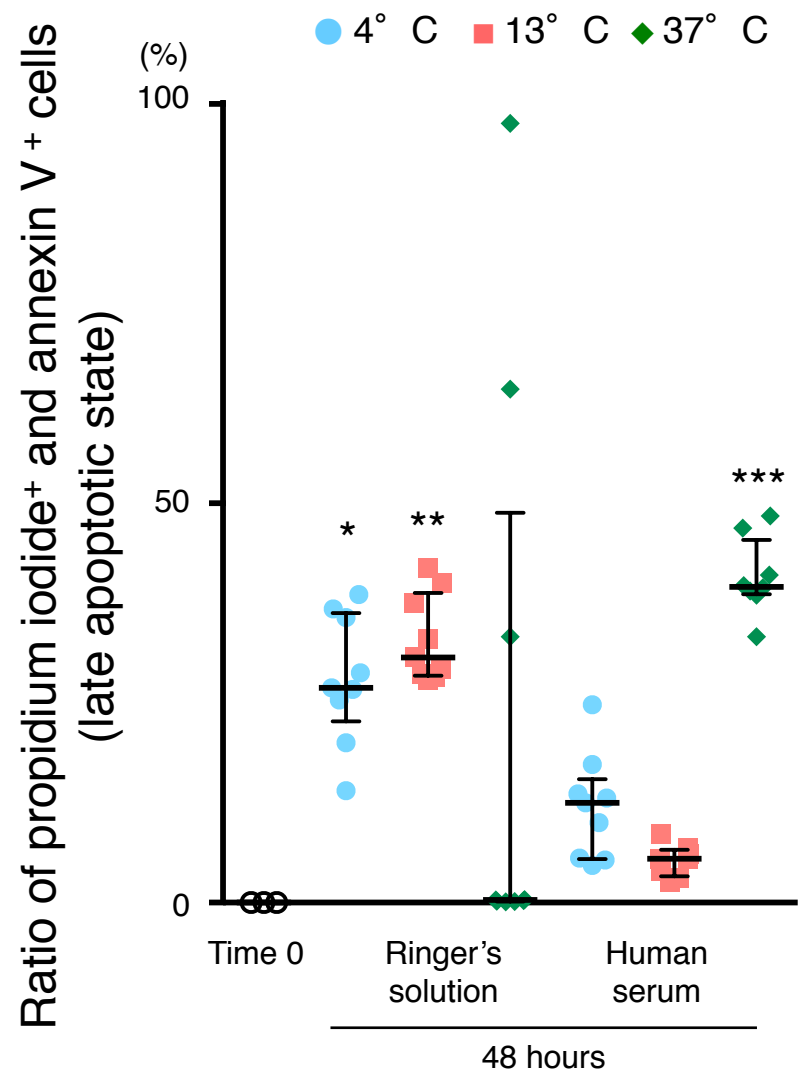

Supplement: Supplementary file 2 — Ratio of propidium iodide positive and annexin V positive synovial MSCs (late apoptotic state). Synovial MSCs before and 48 h after preservation in Ringer’s solution and human serum at each temperature were examined. *p < .05, **p < .01, compared with the value at Time 0 by Friedman test followed by Steel’s multiple comparisons. (PDF 50 kb) [file 13287_2017_596_MOESM2_ESM.pdf]

Supplementary figure 3

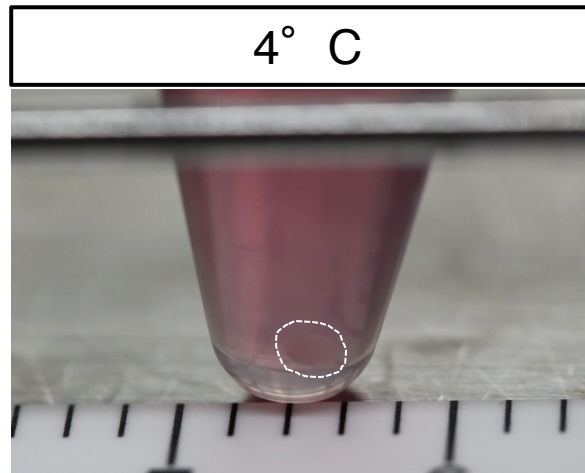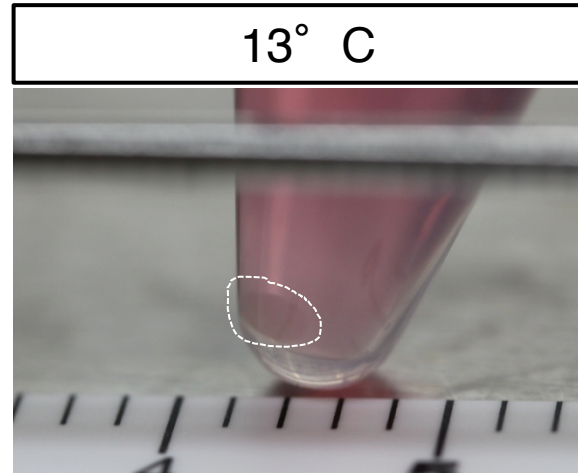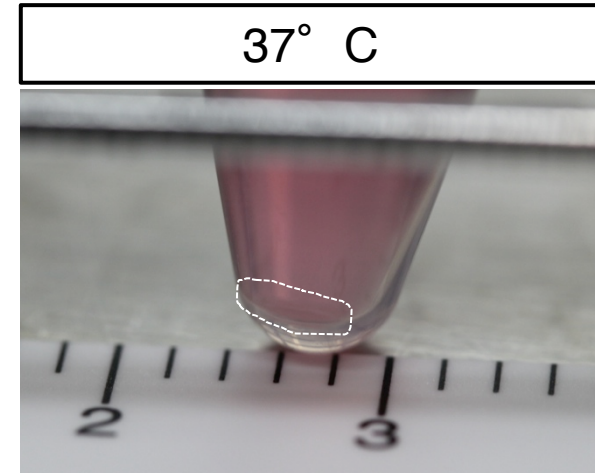

Supplement: Supplementary file 4 — In vitro cartilage formation by pellet culture of synovial MSCs. Synovial MSCs before and 48 h after preservation in human serum at each temperature were pelleted after centrifugation, then cell pellets were cultured in chondrogenic medium for 21 days. Spheroids of cartilage derived from synovial MSCs were observed in cells preserved at 4 and 13 °C, while synovial MSCs were not condensed in cells preserved at 37 °C. Dot line shows cell pellet. (PDF 332 kb) [file 13287_2017_596_MOESM4_ESM.pdf]
