# Supplementary material for: Complete human serum maintains viability and chondrogenic potential of human synovial stem cells: suitable conditions for transplantation
Source: Stem Cell Res Ther. 2017 Jun 13;8:144. doi: 10.1186/s13287-017-0596-0 (PMC5470274; doi:10.1186/s13287-017-0596-0)
Supplement: Supplementary file 3 — Ratio of propidium iodide +/– and annexin V +/– synovial MSCs. Synovial MSCs before and 48 h after preservation in Ringer’s solution and human serum at each temperature were examined (n = 9). Table S2. Wet weight of cartilage pellets derived synovial MSCs. Synovial MSCs before and 48 h after preservation in Ringer’s solution and human serum at each temperature were examined (n = 60). (DOCX 21 kb) [file 13287_2017_596_MOESM3_ESM.docx]

**Supplementary Table 1.**

**Ratio of propidium iodide +/- and annexin V +/- synovial MSCs.** Synovial MSCs before and 48 hours after preservation in Ringer’s solution and human serum at each temperature were examined.

(n=9).

| Temperature conditions |  | Ringer's solution | | Human serum | |
| --- | --- | --- | --- | --- | --- |
|  |  | Median | IQR | Median | IQR |
| 4°C | PI- annexin V- | 57.7 | 9.3 | 82.6 | 10.4 |
|  | PI- annexin V+ | 2.3 | 3.1 | 3.7 | 3.4 |
|  | PI+ annexin V- | 7.3 | 4.7 | 0.8 | 1.0 |
|  | PI+ annexin V+ | 26.9 | 10.3 | 12.5 | 8.0 |
| 13°C | PI- annexin V- | 51.2 | 13.5 | 91.4 | 4.0 |
|  | PI- annexin V+ | 2.7 | 3.5 | 2.7 | 1.3 |
|  | PI+ annexin V- | 13.7 | 7.5 | 0.4 | 0.1 |
|  | PI+ annexin V+ | 30.7 | 9.0 | 5.5 | 2.9 |
| 37°C | PI- annexin V- | 2.6 | 3.8 | 27.5 | 4.9 |
|  | PI- annexin V+ | 94.0 | 30.1 | 19.9 | 11.8 |
|  | PI+ annexin V- | 0.0 | 0.0 | 7.5 | 3.2 |
|  | PI+ annexin V+ | 0.3 | 33.1 | 39.6 | 3.6 |

PI: propidium iodide, IQR: interquartile range.

**Supplementary Table 2.**

**Wet weight of cartilage pellets derived from synovial MSCs.** Synovial MSCs before and 48 hours after preservation in Ringer’s solution and human serum at each temperature were examined (n=60).

|  | | Mean (mg) | | SD | CV | Mean | SD | CV |
| --- | --- | --- | --- | --- | --- | --- | --- | --- |
| Time 0 | | 5.7 | | 1.8 | 30.8% |  |  |  |
| Condition | | Ringer's solution | | | | Human serum | | |
| 48 hours | 4°C | ND | ND | | ND | 4.9 | 3.0 | 61.3% |
|  | 13°C | ND | ND | | ND | 5.5 | 2.4 | 43.9% |
|  | 37°C | ND | ND | | ND | ND | ND | ND |

SD: standard deviation, CV: coefficient of variation, ND: not detected.
